# Looking on the Bright Side Reduces Worry in Pregnancy: Training Interpretations in Pregnant Women

**DOI:** 10.32872/cpe.3781

**Published:** 2021-06-18

**Authors:** Colette R. Hirsch, Frances Meeten, Jill M. Newby, Sophie O’Halloran, Calum Gordon, Hannah Krzyzanowski, Michelle L. Moulds

**Affiliations:** 1 Institute of Psychiatry, Psychology and Neuroscience, King’s College London, London, United Kingdom; 2 School of Psychology, University of Sussex, Brighton, United Kingdom; 3 School of Psychology, University of New South Wales, Sydney, Australia; 4 Black Dog Institute, Hospital Road Randwick, New South Wales, Sydney, Australia; Philipps-University of Marburg, Marburg, Germany

**Keywords:** perinatal mental health, worry, interpretation bias, cognitive bias mediation (CBM), pregnancy, anxiety

## Abstract

**Background:**

Recent evidence suggests that anxiety is more common than depression in the perinatal period, however there are few interventions available to treat perinatal anxiety. Targeting specific processes that maintain anxiety, such as worry, may be one potentially promising way to reduce anxiety in this period. Given evidence that negative interpretation bias maintains worry, we tested whether interpretation bias could be modified, and whether this in turn would lead to less negative thought (i.e., worry) intrusions, in pregnant women with high levels of worry.

**Method:**

Participants (N = 49, at least 16 weeks gestation) were randomly assigned to either an interpretation modification condition (CBM-I) which involved training in accessing positive meanings of emotionally ambiguous scenarios, or an active control condition in which the scenarios remained ambiguous and unresolved.

**Results:**

Relative to the control condition, participants in the CBM-I condition generated significantly more positive interpretations and experienced significantly less negative thought intrusions.

**Conclusions:**

Our findings indicate that worry is a modifiable risk factor during pregnancy, and that it is possible to induce a positive interpretation bias in pregnant women experiencing high levels of worry. Although preliminary, our findings speak to exciting clinical possibilities for the treatment of worry and the prevention of perinatal anxiety.

## Highlights

- Modification of interpretation bias in pregnant women with high levels of worry was examined.
- Participants received interpretation bias training or an active control condition.
- Training led to less negative interpretations and fewer negative thought intrusions.
- Modifying negative interpretation bias in pregnant women may have clinical utility.

The perinatal period, the time from conception to 12 months post birth (Austin, Highet, & Expert Working Group, 2017), is a time of significant change and adjustment. It often brings new stressors which, combined with hormonal fluctuations, can leave women vulnerable to mental health problems. Women are at a higher risk of developing a serious mental illness during the first month postpartum than at any other point in their lives (Stewart et al., 2003), and are also at risk for relapse or recurrence of a pre-existing mental health problem. Perinatal mental health problems are associated with negative outcomes for both mother and baby; for example, poor foetal development (DiPietro et al., 2002), low birth weight (Hedegaard et al., 1993), and greater risk of behavioural, psychological and developmental problems (O’Connor et al., 2002; Stein et al., 2014).

Until relatively recently, most research on perinatal mental health has focused on postnatal depression, with other conditions overlooked (Goodman, Watson, & Stubbs, 2016; Howard, Molyneaux, et al., 2014). In particular, perinatal anxiety has tended to be ignored in favour of depression, despite evidence that anxiety disorders are more prevalent than depression in pregnancy and postpartum (Fairbrother et al., 2016). This is particularly the case in the treatment outcome literature. In a systematic review, Loughnan et al. (2018) identified only one randomised controlled trial evaluating a treatment for perinatal anxiety. With prevalence rates of up to 8.5% (Goodman et al., 2016), and given that maternal prenatal anxiety is associated with a twofold increase in the risk of a child developing psychological disorders (O’Donnell et al., 2014), there is a clear need to develop effective, evidence-based approaches to treat perinatal anxiety.

One promising approach may be to target modifiable psychological processes that maintain anxiety symptoms and their consequences, such as repetitive negative thinking (RNT). RNT refers to types of thinking which are pathological, perseverative and difficult to control (Samtani & Moulds, 2017); for example, worry and rumination. Worry is a form of RNT that is predominantly verbal, difficult to control and involves entertaining potential negative outcomes of future situations (Borkovec, 1994). Rumination primarily involves focusing on events in the past, as well as one’s perceived personal inadequacies, current mood/symptoms and their causes and consequences (Nolen-Hoeksema, 1991). Both these forms of RNT are experienced as unwanted negative intrusive thoughts that come to mind unbidden, and capture attention such that it is difficult to shift focus away from the thought. Moulds et al. (2018) proposed that RNT could be an important factor to target in interventions to improve perinatal distress. In keeping with this, a recent study of pregnant women (Hirsch, Meeten, et al., 2020) demonstrated that worry and RNT more generally was associated with increased levels of perinatal anxiety and depression. The predictive role of worry in the development and maintenance of anxiety is well-established, and recent research has indicated that this may similarly apply in the perinatal context. For example, Schmidt et al. (2016) reported that levels of worry in the first four months of pregnancy predicted anxiety and depression symptoms in the third trimester.

One key cognitive process proposed to contribute to pathological worry is negative interpretation bias: the transdiagnostic tendency to perceive ambiguous information or events as threatening or negative (Hirsch & Mathews, 2012; Hirsch et al., 2016). Krahé et al. (2019) found that greater levels of negative interpretation were associated with increased worry. Similarly, Hirsch, Meeten, et al. (2020) demonstrated that higher levels of both worry and anxiety in pregnant women are associated with more negative interpretation bias. These findings speak to the clinically related possibility that modifying interpretation bias may reduce worry. One experimental methodology showing promise in this regard is cognitive bias modification for interpretation (CBM-I).

The goal of CBM-I is to facilitate consistent generation of positive interpretations of ambiguous information (where the interpretation could be positive or threatening) via repeated computerised practice. Specifically, participants listen to ambiguous scenarios, with ambiguity being resolved by the final word in a benign manner (see Appendix A in the Supplementary Materials for an example scenario). Evidence indicates that a single session of CBM-I can modify interpretation bias and in turn reduce worry in high worriers (Feng et al., 2020; Hirsch et al., 2009), as well as those with generalised anxiety disorder (GAD) (Hayes, Hirsch, Krebs, & Mathews, 2010). In another GAD sample, Hirsch et al. (2018) demonstrated that multi-session positive CBM-I training resulted in a more positive interpretation bias and reduced worry and anxiety one month later compared to an active control condition. More recently, community participants with high levels of RNT (worry and/or rumination) completed an enhanced version of CBM-I where participants were instructed to generate positive resolutions to ambiguous scenarios (rather than be presented with a positive resolution) for half of the scenarios, in order to aid generalisation and engagement. Participants were also instructed to generate positive images of the outcome for each scenario. This led to more positive interpretation bias, fewer negative interpretations, and lower levels of RNT, anxiety and depression, relative to a control condition in which ambiguity was unresolved (Hirsch, Krahé, Whyte, Bridge, et al., 2020). These findings prompt the clinically interesting possibility that CBM-I can be used as a potential intervention for anxiety.

To determine whether CBM-I can help reduce worry and anxiety via a web-based platform with no face-to-face contact with researchers during assessment or training, we conducted a study with a sample of individuals with GAD with or without comorbid major depressive disorder (Hirsch, Krahé, Whyte, Krzyzanowski, et al., 2020). Training was highly effective at reducing negative interpretations compared to the control condition. Importantly, reductions in worry, rumination, anxiety and depression were evident at three-months follow-up. Furthermore, effects were mediated by changes in interpretation bias. These findings raise the possibility of CBM-I forming a low-intensity intervention for pregnant women at risk of escalating levels of anxiety or depression due to heightened RNT. As an online intervention, it could be completed at a location and time convenient for pregnant women, and thus has scope to be more readily integrated into daily life.

The possibility that CBM-I may have utility in facilitating a more positive interpretation bias in pregnant women who engage in high levels of worry remains untested. Given that pregnant women who worry have a more negative interpretation bias (Hirsch, Meeten, et al., 2020), and the proposal that targeting RNT, such as worry, in pregnancy may have the potential to prevent and treat postpartum anxiety (Moulds et al., 2018), testing whether CBM-I can shift interpretive bias in pregnant high worriers represents a logical first step. Accordingly, we recruited pregnant women with self-reported high levels of worry who were randomly allocated to either (i) CBM-I (i.e., interpretation training enhanced with positive imagery and self-generation) or (ii) control (no resolution of ambiguity nor positive imagery) conditions. We hypothesised that participants in the CBM-I condition would generate more positive interpretations and thus demonstrate a positive interpretation bias compared to those in the control condition. We also hypothesised that participants in the CBM-I condition would experience fewer negative thought intrusions (indicative of worry) during a behavioural worry task in which they were instructed to focus on their breathing, relative to participants in the control condition.

## Method

### Study Registration

The study was registered on Open Science Framework: https://osf.io/ye84g. See Appendix B in the Supplementary Materials for registered information.

### Participants

49 women with high levels of self-reported worry (scoring ≥ 56^1^1In a sample of individuals diagnosed with GAD, a PSWQ score of 56 was one standard deviation below the mean (Molina & Borkovec, 1994) and is commonly used as a cut-off in research (Feng et al., 2020; Hirsch, Perman, et al. 2015). Accordingly, we classified participants as high worriers if their PSWQ score was ≥ 56. on the Penn State Worry Questionnaire cf. Hayes, Hirsch, & Mathews, 2010) completed the study and 47 women completed useable data (see Table 1 for demographic information). Participants were required to be 16 or more weeks pregnant, fluent in English, with normal or corrected vision and hearing, and have no history of either stillbirth or three or more miscarriages. Participation involved attending a session in the lab, and participants were reimbursed £25 for taking part.

**Table 1:** Mean Demographic and Statistics Characteristics and Questionnaires (Standard Deviation in Parenthesis)

Characteristic	CBM-I *N* = 23	Control *N* = 24	*t*(45)	*p*
**Age**	33.35 (4.78)	32.46 (4.65)	0.65	0.52
**Weeks of gestation**	26.96 (7.10)	28.29 (6.62)	0.67	0.51
**PSWQ**	64.30 (5.67)	66.13 (5.66)	1.10	0.28
**RTQT**	39.70 (10.63)	40.67 (7.01)	0.37	0.71
**PASS**	43.09 (15.83)	47.54 (17.87)	0.90	0.37
**EDPS**	11.87 (3.55)	14.21 (5.37)	1.76	0.09
**PHQ-9**	8.87 (3.88)	11.00 (6.09)	1.42	0.16
**GAD-7**	8.52 (4.12)	11.42 (5.36)	2.07	0.04
**RRS**	54.48 (13.30)	52.63 (13.54)	0.47	0.64

*Note*. CBM-I = cognitive bias modification for interpretation; Weeks of gestation = number of weeks pregnant at time of testing; PSWQ = Penn State Worry Questionnaire; RTQT = Trait Repetitive Thinking Questionnaire; PASS = Perinatal Anxiety Screening Scale; EPDS = Edinburgh Postnatal Depression Scale; GAD7 = Generalised Anxiety Disorder Questionnaire; PHQ9 = Patient Health Questionnaire; RRS = Ruminative Response Scale.

Individuals who expressed interest in the study were sent a screening questionnaire via Qualtrics, an online data acquisition platform. 163 women completed the screening questionnaire, of whom 64 did not meet the inclusion criteria. 99 respondents were eligible to take part in the study and were invited via email to take part in the study. 63 of these responded and were offered a testing date. Of these, 49 participants completed the study, while six were found to be ineligible on the day of testing due to their Penn State Worry Questionnaire score (Meyer et al., 1990) being below cut off, seven withdrew before attending and one session was cancelled due to the COVID-19 pandemic. Two participants’ data was not included in the study as their responses to the Recognition Test Comprehension Questions indicated they had either not understood or not engaged with the task. The final sample of 47 participants were aged between 22 and 42 years (*M* = 32.89, *SD* = 4.69), and ranged between 16 and 39 weeks pregnant (*M* = 27.64, *SD* = 6.82). 12 participants had one child and two participants had two children. The other 35 participants were pregnant with their first child.

### Sample Size

An a-priori power calculation with an alpha of .05 and power of .80 was computed in GPower. The effect size was determined by a study examining the effects of interpretation bias manipulation on the Recognition Test (Feng et al., 2020). Projected sample size was 26 per condition. As we did not know whether pregnancy would influence the capacity to modify interpretation bias, we elected to increase the planned number of participants recruited per condition to 30. However, due to the COVID-19 pandemic in 2020, face-to-face testing was ultimately prohibited. Recruitment and testing ended prematurely after testing 49 participants (two participants were excluded due to performance on the Recognition Test) resulting in final samples of *n* = 23 and *n* = 24 in the CBM-I and control conditions, respectively.

### Measures and Materials

#### Questionnaires

##### Penn State Worry Questionnaire (PSWQ)

The PSWQ (Meyer, Miller, Metzger, & Borkovec, 1990) consists of 16 statements related to worry (e.g., *My worries overwhelm me*) which are rated from 1 (*not at all typical of me*) to 5 (*very typical of me*). The PSWQ has high internal consistency (present sample Cronbach’s α = .70), convergent and discriminant validity (Brown, Antony, & Barlow, 1992), and good test-retest reliability (Meyer et al., 1990).

##### Other standardised questionnaires

Perinatal anxiety was assessed using the Perinatal Anxiety Screening Scale (PASS; Somerville et al., 2014; Cronbach’s α = .94 in current sample). Perinatal depression was assessed with the Edinburgh Postnatal Depression Scale (EPDS; Cox, Holden, & Sagovsky, 1987: Cronbach’s α = .84). General depressed mood was assessed using the Patient Health Questionnaire 9 (PHQ-9, Kroenke & Spitzer, 2002; Cronbach’s α = .84) and anxiety symptoms using the Generalized Anxiety Disorder 7-item scale (GAD-7; Spitzer et al., 2006; Cronbach’s α = .87). Trait RNT was assessed with the Repetitive Thinking Questionnaire (RTQ-T [trait]; McEvoy, Thibodeau, & Asmundson, 2014; Cronbach’s α = .90). Ruminative Response Scale (RRS; Nolen-Hoeksema & Morrow, 1991; Cronbach’s α = .93) was used to assess depressive rumination^2^2VAS mood ratings were also taken during the study, but were not available for analysis due to the university being closed because of COVID-19..

#### Tasks

##### Worry induction

Participants identified a current worry topic (related to their pregnancy or other aspects of their life) and were asked a series of questions to prime salient features. They were instructed to silently worry about this topic as they normally would for five minutes.

##### Interpretation assessment task - Recognition Test

The first phase of this task (Hirsch et al., 2018; adapted from Mathews & Mackintosh, 2000) requires participants to read a series of ambiguous scenarios. The last word of each scenario (which leaves the ambiguity unresolved) is presented as a word fragment, and participants are instructed to fill in the first missing letter of that word. Next, participants complete a comprehension question (yes/no) about the scenario (see Appendix A in the Supplementary Materials for example). In the second phase, participants are presented with a scenario title and four statements in random order, then indicate how similar each statement is to the meaning of the original scenario. The statements include one positive target (in keeping with the positive interpretation of the original scenario), one negative target, one positive and one negative foil unrelated to the scenario meaning. Participants rate each statement on a scale from 1 (*very different in meaning*) to 4 (*very similar in meaning*). Interpretation bias is assessed by calculating a positivity index, which is calculated by subtracting the mean ratings for negative targets from the mean ratings for positive targets. Higher scores indicate a more positive interpretation bias.

##### Breathing Focus Task

In the version of the task (Feng et al., 2020; adapted from Ruscio & Borkovec, 2004) employed in this study, participants first practiced the breathing focus task. Next, they were instructed to engage in worry about a current worry topic for five minutes, then completed a five-minute breathing focus task. During this task, participants were instructed to focus on their breathing. They were given a series of prompts (12 computerised tones) throughout the task; at each prompt, participants were asked to indicate if they were focusing on their breathing as instructed, or if their mind had wandered to another topic (i.e., they were experiencing a thought intrusion). If the latter, participants were asked to indicate the valence of the intrusion (i.e., positive, negative or neutral). Negative thought intrusions are interpreted to be indicative of worry, as per previous CBM-I studies (e.g., Feng et al., 2020).

#### CBM-I Condition

##### Imagery Practise Task

Participants in the CBM-I condition completed an online imagery practice task (adapted from Holmes et al. (2006) and used in Hirsch, Krahé, Whyte, Bridge, et al., 2020; Feng et al., 2020) to help them generate vivid mental images, and to instruct them on how to hold them in mind (see Feng et al., 2020).

##### Cognitive Bias Modification for Interpretation (CBM-I)

CBM-I is a scenario-based task that requires participants to listen (over headphones) to 40 scenarios which present common worry-related situations that are initially emotionally ambiguous. Participants in the active condition were provided with a positive resolution (i.e. ending) of the ambiguous scenario for 20 trials, and instructed to generate their own positive resolution for the 20 remaining trials. Participants are instructed to use mental imagery to vividly picture the resolution. After each scenario, participants are presented with a ‘Yes/No’ comprehension question, designed to emphasise the desired interpretation of the scenario. They then receive feedback (‘correct/incorrect’) on these answers. Participants then rate the positivity of the scenario, on a scale of 0 (‘*not at all’)* to 100 (*‘extremely’)* (see Appendix A, Supplementary Materials, for example).

#### Control condition

##### Filler Task

The Feng et al. (2019) filler task was used to match the time taken to complete the imagery training in the CBM-I condition.

##### Sham Training

Similar to CBM-I training, participants listened to 50 ambiguous worry-related scenarios over headphones. An increased number of trials was required to match the duration of CBM-I training. In this condition ambiguity remained unresolved, and participants were not instructed to generate particular outcomes. Participants completed comprehension questions without feedback, thus allowing for either positive or negative interpretations without correction.

### Procedure

**Figure 1:**
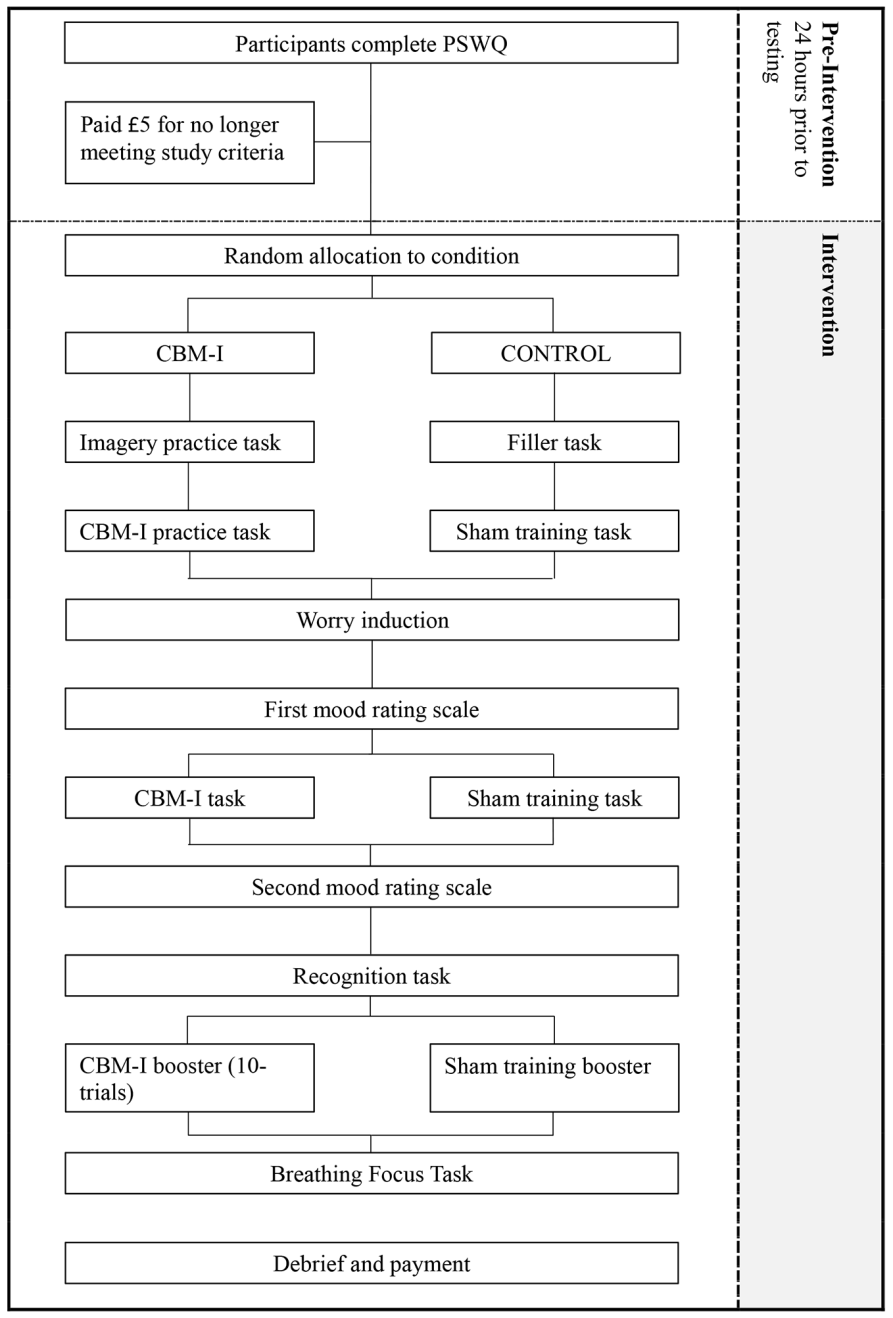
Overview of Study Procedure

Participants completed the PSWQ online within the 24 hours prior to the experimental testing session, to ensure that they met study eligibility criteria. Before coming into the lab, participants were randomly allocated to the CBM-I or control condition on the basis of an allocation by an independent researcher. They then completed the study tasks associated with their allocated condition. See Figure 1. for an overview of the study procedure.

## Results

### Questionnaire Measures for CBM-I and Control Conditions

See Table 1 for means of questionnaire measures and statistics for participants included in the analysis. The only significant between-condition difference to emerge was for GAD-7; such that participants in the control condition reported higher anxiety. Importantly, however, we note that the conditions did not differ on the PASS, – i.e., a measure of perinatal anxiety specifically (rather than a measure of general anxiety developed for non-pregnant populations).

### Assessing the Impact of CBM-I on Interpretation Bias (Hypothesis 1)

To examine the effect of condition on interpretation bias, we conducted a regression analysis with mean positivity index score as the dependent variable. Condition^3^3As GAD7 scores were significantly different at baseline we re-ran the regression analysis with mean centred GAD7 scores and an interaction variable of (mean centred) GAD7 and condition. Neither GAD7 scores (*p* = .67) or the interaction term (*p* = .54) were significant predictors in the model. Condition remained a significant predictor (*p* = .02). significantly predicted post-training positivity index score, *b* = 0.54, *SE* = .19, *p* = .007, 95% CIs [0.16, 0.92]. The mean positivity index was higher for the CBM-I (*M* = 0.35, *SD* = 0.64) than the control (*M* = 0.19, *SD* = 0.65) condition, confirming that CBM-I was effective in facilitating a positive interpretation bias.

### Assessing the Impact of CBM-I on Negative Thought Intrusions (Hypothesis 2)

To examine the effect of condition on negative thought intrusions, we conducted a bootstrapped (due to non-normality of data) regression analysis with number of negative thought intrusions from the breathing focus task as the dependent variable. Condition significantly predicted post-training positivity index score, *b* = -1.11, *SE* = .45, *p* = .02, 95% CIs [-1.96, -0.28]. Consistent with the hypothesis, participants in the CBM-I condition reported significantly fewer intrusions (*M* = 1.50, *SD* = 1.01) than did those in the control condition (*M* = 2.61, *SD* = 1.85).

## Discussion

In this first study of interpretation training in pregnant worriers, we successfully induced a positive interpretation bias using CBM-I. Consistent with Hirsch et al. (2009) and Feng et al. (2019), participants in the CBM-I condition reported fewer negative thought intrusions relative to the control condition, supporting a causal role for interpretation bias in maintaining worry in pregnant women. As the first study to employ CBM-I to test questions about interpretation bias and worry in pregnant women, our results extend the CBM literature in important ways. First, on a methodological note, they demonstrate the applicability and effectiveness of CBM-I in the perinatal context. Second, they confirm that interpretation bias maintains worry in pregnant women. Whilst this relationship is well-established in the broader literature (Feng et al., 2019; Hirsch et al., 2009; Hirsch, Krahé, Whyte, Bridge, et al., 2020) given the unique and multi-faceted circumstances and changes (e.g., biological, cognitive) which characterise the perinatal period, our results are theoretically important in confirming this link in a perinatal sample.

Third, by indicating that worry is a modifiable psychological risk factor in pregnancy, our findings have clinical promise. As noted earlier, the treatment of perinatal anxiety has received limited research attention. Further, the treatments that have been developed are primarily generic such that they are comprised of standard CBT techniques, including challenging cognitions by generating alternative interpretations (e.g., Forsell et al., 2017; see Moulds et al., 2018). In contrast, CBM-I seeks to enhance access to positive interpretations in a more direct, automatic way. Our findings suggest that developing novel approaches which draw on experimental findings and directly target factors that have been identified to maintain anxiety (e.g., worry) to potentially supplement existing treatment approaches may be a promising future clinical direction.

Moreover, our findings speak to the issue of prevention. Given growing evidence that antenatal RNT predicts perinatal mental health problems (DeJong et al., 2016; Schmidt et al., 2016), the prospect of reducing worry in pregnant women by targeting interpretation bias represents an exciting possibility for preventing postpartum anxiety. Topper et al. (2017) found that that a preventive intervention which targeted RNT reduced the onset of depression and anxiety 12 months later. Our finding that antenatal worry is a modifiable risk factor similarly raises the possibility that an intervention targeting worry may also have utility in preventing subsequent mental health problems in the postnatal period.

We acknowledge some limitations and suggest future research directions. First, while single-session CBM experiments critically advance understanding of theoretical mechanisms, they do not provide sufficient evidence regarding the sustained consequences of targeting interpretation bias in this way (Hirsch et al., 2018). However, we note that recent studies using multiple CBM-I sessions (e.g., 10 internet-delivered sessions) have reported encouraging preliminary evidence of the longevity of effects (i.e., reductions in RNT at one-month follow-up; Hirsch et al., 2018; Hirsch, Krahé, Whyte, Bridge, et al., 2020). Future research employing multiple sessions with an extended follow-up period is needed before conclusions can be drawn about potential clinical benefit and preventive utility in the perinatal context. Second, we did not gather detailed information about previous numbers of miscarriages or complications in participants’ current (or any previous) pregnancy, leaving it unknown whether our findings generalise to pregnant women who have experienced pregnancy loss or complications in participants’ current (or any previous) pregnancy.

Third, we did not assess interpretation bias or the presence of negative intrusions pre-training, and thus do not know whether groups differed at the outset. However, participants were randomised to condition by a researcher outside of the study team, making these possible explanations for the results unlikely. Fourth, randomisation led to differences in anxiety (GAD-7) between groups. Finally, due to COVID-19 pandemic ruling out completion of data collection, the number of participants was slightly below that recommended in the original sample size calculation.

Our findings raise interesting possibilities for future research. In a recent fully web-based study, Hirsch, Krahé, Whyte, Krzyzanowski, et al. (2020) reported that CBM-I led to reductions in depression and anxiety, as well as worry and rumination, in participants with GAD with or without comorbid depression. The effects persisted to 3-month follow-up, and notably, were mediated by changes in interpretation bias. These results raise the exciting possibility that CBM-I could form a low intensity intervention to treat or prevent anxiety and worry, with potential for application in the perinatal context. Further, given evidence that CBM-I may be effective in modifying interpretation bias in the context of a range of mental health conditions (e.g., depression, Hirsch et al., 2018; eating disorders, Turton et al., 2018; social anxiety, Stevens et al., 2018), another potential research direction could be to investigate the effectiveness of CBM-I for other perinatal psychological symptoms, beyond anxiety.

In sum, this study is the first to evaluate the effectiveness of single session CBM-I for reducing worry in pregnant women. Our findings provide empirical support for interpretive bias as a mechanism underlying antenatal worry, and thus indicate that worry is a modifiable risk factor during pregnancy. Future research with a broader sample warrant investigation (where the current sample were from South London and had not experienced three or more miscarriages) to determine if findings generalise to a more heterogenous sample. Furthermore, future research with pregnant women diagnosed with GAD is needed to confirm that these results are generalisable to treatment-seeking, clinical samples. Nonetheless, given evidence that worry early in pregnancy predicts later anxiety, these data represent an important first step in investigating whether CBM-I holds promise as a therapeutic approach to address perinatal mental health problems.

## Supplementary Materials

The following Supplementary Materials are available (for access see Index of Supplementary Materials below):

- Via the Open Science Framework (OSF) repository: The preregistration for the study
- Via the PsychArchives repository: Supplementary Materials (Appendices)
  - Appendix A includes: Further methodological details of cognitive bias modification for interpretation and the Recognition Task assessment of interpretation bias
  - Appendix B includes: Open Science Framework pre-registered study protocol

10.23668/psycharchives.4856 Supplement 1 Supplementary materials to "Looking on the bright side reduces worry in pregnancy: Training interpretations in pregnant women" [Appendix A]

10.23668/psycharchives.4856 Supplement 2 Supplementary materials to "Looking on the bright side reduces worry in pregnancy: Training interpretations in pregnant women" [Appendix B]

HirschC. R.
MeetenF.
NewbyJ. M.
O’HalloranS.
GordonC.
KrzyzanowskiH.
MouldsM. L.
 (2018). *Cognitive Bias Modification for Interpretation (CBM-I) to reduce worry in pregnant women*
[Preregistration]. PsychOpen. https://osf.io/ye84g


HirschC. R.
MeetenF.
NewbyJ. M.
O’HalloranS.
GordonC.
KrzyzanowskiH.
MouldsM. L.
 (2021). Supplementary materials to "Looking on the bright side reduces worry in pregnancy: Training interpretations in pregnant women"
[Appendices]. PsychOpen. 10.23668/psycharchives.4856
PMC966712836397954
