# Supplementary material for: Looking on the Bright Side Reduces Worry in Pregnancy: Training Interpretations in Pregnant Women
Source: Clin Psychol Eur. 2021 Jun 18;3(2):e3781. doi: 10.32872/cpe.3781 (PMC9667128; doi:10.32872/cpe.3781)
Supplement: Supplement 1 [file cpe-03-3781-s01.pdf]

Appendix A

Looking on the bright side reduces worry in pregnancy: training interpretations in pregnant women

Hirsch, C. R*. [1], Meeten, F*.[2], Newby, J. M.[3,4], O'Halloran, S.[1], Gordon, C.[1], Krzyzanowski, H.[1] & Moulds, M. L.[3]

* Joint 1st author

affiliations

[1]Institute of Psychiatry, Psychology and Neuroscience, King's College London, UK

[2]School of Psychology, University of Sussex, UK

[3]School of Psychology, University of New South Wales, Sydney, Australia

[4]Black Dog Institute, Hospital Road Randwick, New South Wales, Sydney, Australia

Appendix A

1. **Cognitive Bias Modification for Interpretation (CBM-I; Hirsch et al. 2009; Hirsch, Krahé, Whyte, Bridge, et al. 2020)**

CBM-I is an online, scenario-based task that requires participants to listen (over headphones) to 40 scenarios which present common worry-related situations that are initially emotionally ambiguous. Participants in the active condition were provided with a positive resolution (i.e., ending) of the ambiguous scenario for 20 trials, and are instructed to generate their own positive resolution for the 20 remaining trials. Participants are instructed to use mental imagery to vividly picture the resolution. After each scenario, participants are presented with a 'Yes/No' comprehension question, designed to emphasise the desired interpretation of the scenario. They then receive feedback ('correct/incorrect') on these answers. Participants then rate the positivity of the scenario, on a scale of 0 (*'not at all')* to 100 (*'extremely')*.

Example scenario: You are getting a piece of work back from your supervisor today. When working on it, you were very busy with other tasks but spent as much time on it as you could manage. When you read your supervisor's feedback, you understand that your work was fine.

Comprehension question: Is your supervisor pleased with your work?

Answer: Yes

2. Interpretation assessment task - Recognition Task (Hirsch et al., 2018; adapted from Mathews & Mackintosh, 2000)

<u>Example Scenario</u>

Title: The car park

It is late at night and you are in a multi-storey car park trying to find your car. You have been looking for about ten minutes and still cannot find it. You hear a noise behind you and see a shadow of

Som_th_ng [something].

Question: Did you find your car right away? (Correct answer: no)

In the second phase, participants are presented with a scenario title and four statements in random order, and then asked to indicate how similar each statement was to the meaning of the original scenario. The statements include one positive target (in keeping with the positive interpretation of the original scenario), one negative target, and one positive and one negative foil unrelated to the scenario meaning. Participants rate each statement on a scale of 1 (*very different in meaning*) to 4 (*very similar in meaning*). For example, for the carpark scenario:

- You see a security person approaching to help you (positive target)
- You see someone coming towards you looking threatening (negative target)
- You see some money on the floor and pick it up (positive foil)
- You see that you have forgotten your ticket and will have to pay a fine (negative foil)
