# Supplementary material for: Looking on the Bright Side Reduces Worry in Pregnancy: Training Interpretations in Pregnant Women
Source: Clin Psychol Eur. 2021 Jun 18;3(2):e3781. doi: 10.32872/cpe.3781 (PMC9667128; doi:10.32872/cpe.3781)
Supplement: Supplement 2 [file cpe-03-3781-s02.pdf]

Appendix B

# Looking on the bright side reduces worry in pregnancy: training interpretations in pregnant women

Hirsch, C. R*. [1], Meeten, F*.[2], Newby, J. M.[3,4], O'Halloran, S.[1], Gordon, C.[1], Krzyzanowski, H.[1] & Moulds, M. L.[3]

* Joint 1st author

affiliations

[1]Institute of Psychiatry, Psychology and Neuroscience, King's College London, UK

[2]School of Psychology, University of Sussex, UK

[3]School of Psychology, University of New South Wales, Sydney, Australia

[4]Black Dog Institute, Hospital Road Randwick, New South Wales, Sydney, Australia

Appendix B

**Pre-registered protocol**

Title:
Cognitive Bias Modification for Interpretation (CBM-I) to reduce worry in pregnant women

Authors:
Colette Hirsch, Calum Gordon

Research Questions:
This experiment is designed to answer two questions:

1) Can a single session of cognitive bias modification for interpretation (CBM-I) promote more positive interpretations, compared to an active control condition, in pregnant women?

2) If a more benign interpretation bias is found after CBM-I training, does this lead to fewer negative thought intrusions, compared to an active control condition?

Hypotheses:
Our hypotheses are directional.

Hypothesis 1: We predict that CBM-I, relative to the control condition, will increase positive interpretations following the single session intervention.

Hypothesis 2: We predict that CBM-I, relative to the control condition, will be associated with fewer self-reported negative intrusions on a Breathing Focus task

## Sampling Plan

Existing data:
Registration prior to the creation of data

Explanation of existing data:
There is no existing data for this study

Data collection procedures:
Participants will be recruited via the internet e.g., through adverts on social media platforms (e.g., Facebook), and classified advert websites (e.g. Gumtree). Advertisement opportunities will also be sought with local, pregnancy relevant groups, such as antenatal classes, and in local mother & baby shops. Participants will receive an incentive of £25 for taking part in the research.

Participants (aged 18 or over) will be eligible to participate in the study if they are 22-28 weeks pregnant, and have not previous experienced a stillbirth. Since we are looking to recruit high worriers for this study, participants will need to have a score on the Penn State Worry Questionnaire (PSWQ) >61 at screening. This same questionnaire will be administered 24hrs before the scheduled testing session, and participants will be screened again, with scores needing to be >56 for further eligibility. Given the study involves reading words and listening to scenarios, participants will need to be fluent in reading and listening to English, and have normal, or corrected to normal, vision and hearing.

Potential participants who indicate their interest in response to our adverts will be contacted, and sent a brief screening questionnaire to assess for the above criteria. Those who are deemed eligible will be scheduled for testing. To ensure that we are only testing high-worriers, participants will be resent the PSWQ to be completed online within 24hrs of their scheduled appointment. Any participants who have fallen below cut-off at this point will be deemed ineligible and will be offered £5 in vouchers as a thank you for their time thus far. In the same survey, participants will also complete a number of

other questionnaires, including the Perinatal Anxiety Screening Scale, Edinburgh Depression Scale, Repetitive Negative Thinking Questionnaire-10, Ruminative Response Subscale, Generalised Anxiety Disorder 7-item scale, and Patient Health Questionnaire, as well as a number of demographic questions, such as number of children and relationship status.

Eligible participants will attend a lab session and be randomly allocated to either the CBM-I or control condition. If a participant is in the CBM-I condition, they will complete a short imagery practice , whilst those in the control condition will complete a filler task. After this, participants will be asked to identify a recent worry and worry about this for 5 mins, before completing either the CBM-I or Control training. Following CBM-I/control training, participants will do a short filler task, before completing the recognition test, assessing interpretation bias. Participants will then run through a short CBM-I or Control booster training, before completing a Breathing Focus task designed to measure negative intrusions. Participants will also fill in 2 mood rating forms across the testing session.

The study is anticipated to run between December 2018 and December 2019.

Sample size:
Our projected number of participants is 60

Sample size rationale:
The project sample size of 60 is based on power 0.8 and alpha 0.05, using effect sizes from research on the breathing focus task in post-modification assessment (Hayes et al., 2010; Hirsch et al., 2009), which is 24 participants per group. Another 6 participants are added to each group as it is unclear whether effects will be as evident in this population

Stopping rule:
N/A

# Variables

## Manipulated variables:

We will manipulate interpretation bias by training one group (those in the CBM-I condition) to make more positive interpretations using a scenario-based cognitive bias modification paradigm (see Krahé, Mathews, Whyte, & Hirsch, 2016; Hirsch et al., under review).

In keeping with Hirsch et al. (under review), the CBM-I training will involve listening to some ambiguous scenarios over headphones. Participants are either presented with positive outcomes for a scenario and asked to generate a vivid mental image of it (50% of trials), or are asked to generate their own positive outcome and vividly imagine it (50% of trials). Pre CBM-I training, participants will be given some practice in generating mental images. After each scenario, participants will be asked a comprehension question, which reinforces the intended positive interpretation.

The control group will complete a training that is similar in content, but does not generate or reinforce positive outcomes. Participants will be presented with ambiguous scenarios, but the ambiguity will remain unresolved. As a result, interpretation bias should remain unchanged for this group. They will also be asked comprehension questions. Instead of the imagery practice, participants in the control condition will complete a neutral filler task (watching a video on an unrelated subject and answering questions about it) before taking part in the scenario-based training.

Measured variables:
*Describe each variable that you will measure. This will include outcome measures, as well as any predictors or covariates that you will measure. You do not need to include any variables that you plan on collecting if they are not going to be included in the confirmatory analyses of this study.*

Appendix B

Interpretation bias measures

Interpretation bias will be assessed using a Recognition Test (Mathews & Mackintosh, 2000). This involves reading a series of ambiguous descriptions in which the ambiguity is not resolved. Some of these materials present ambiguous situations in everyday life where one could worry, and some present ambiguous situations that someone might encounter during pregnancy. Subsequent recognition ratings of possible interpretations will test which interpretations participants generated (i.e. positive or negative). During the encoding phase, 21 ambiguous descriptions of situations will be presented, and participants will be asked a comprehension question. During the recognition phase to assess interpretations, a list of different possible interpretations will be presented, and participants are required to rate from 1 (very different in meaning) to 4 (very similar in meaning) as to how related the meaning is to the original scenario.

Primary symptom measures of worry, RNT and anxiety

In The Breathing Focus task (adapted from: Hirsch, Hayes, & Mathews, 2009), participants will be asked to worry for five minutes, and then focus on their breathing for five minutes. During this focusing period, 12 audio cues, presented at random intervals, will prompt participants to indicate whether they are focusing on breathing or they have thought intrusions that are positive, negative or neutral. At the end of the breathing focus period, participants will complete brief mood rating scales and rate their level of focus. The number of intrusions during breathing focus period will be calculated. An expanded descriptions component will be used, where participants will be asked to expand on any intrusions sampled during the Breathing Focus task. These will be audio recorded (full anonymity maintained, verbal consent to be included in recording) and will be rated for valence content by a raters who are blind to the research question.

Participants will also complete mood ratings after the first worry induction (pre-CBM-I), and before the recognition test (post-CBM-I)

The following Standardised self-report questionnaires will be delivered before the session: Perinatal Anxiety Screening Scale (PASS; Somerville et al., 2014) - measures anxiety symptoms throughout the perinatal period; Edinburgh Postnatal Depression Scale (EPDS; Cox, Holden, & Sagovsky, 1987) - indexes symptoms of perinatal and postnatal depression; Repetitive Thinking Questionnaire-10 (RTQ-10; McEvoy, Mahoney, & Moulds, 2010) – a transdiagnostic validated measure of the tendency to engage in RNT. Penn State Worry Questionnaire will assess trait worry (Meyer, Miller, Metzger, & Borkovec, 1993); while depressive rumination will be assessed by Response Style Questionnaire Ruminative Response Subscale (RRS; Butler, & Nolen-Hoeksema, 1994). Generalized Anxiety Disorder 7-item scale (GAD-7; Spitzer, Kroenke, Williams, & Löwe, 2006) - screens and measures severity of GAD; Patient Health Questionnaire (PHQ-9; Kroenke, Spitzer, & Williams, 2001) - screens and measures severity of depression.

Indices:
*If any measurements are going to be combined into an index (or even a mean), what measures will you use and how will they be combined? Include either a formula or a precise description of your method. If your are using a more complicated statistical method to combine measures (e.g. a factor analysis), you can note that here but describe the exact method in the analysis plan section.*
Total scores will be calculated for all self-report questionnaires described above. The outcome variable of the recognition test is an index of mean similarity ratings for positive targets minus mean similarity ratings for negative targets (as in Hirsch et al., 2018).

Design Plan
Study Type:
Experiment - A researcher randomly assigns treatments to study subjects, this includes field or lab experiments. This is also known as an intervention experiment and includes randomized controlled trials.

Appendix B

Blinding:
Participants will be blind to which condition they have been assigned

Study design:
*Describe your study design. Examples include two-group, factorial, randomized block, and repeated measures. Is it a between (unpaired), within-subject (paired), or mixed design? Describe any counterbalancing required. Typical study designs for observation studies include cohort, cross sectional, and case-control studies.*
This study will employ a two-group, between-subjects design. The between-subjects factor is condition (2 levels: CBM-I, control).

Randomisation:
*If you are doing a randomized study, how will you randomize, and at what level?*
We will randomise which of the two conditions (CBM-I or control) participants are allocated to, by means of a random allocation sequence. Allocation concealment will be implemented so that experimenters are not aware of which condition the next participant will be assigned to.

Analysis Plan
Statistical Models:
*What statistical model will you use to test each hypothesis? Please include the type of model (e.g. ANOVA, multiple regression, SEM, etc) and the specification of the model (this includes each variable that will be included as predictors, outcomes, or covariates). Please specify any interactions that will be tested and remember that any test not included here must be noted as an exploratory test in your final article.*
Regression analyses (with bootstrapped standard errors in the event of non-normally distributed data) will be used to test the hypothesis that the CBM-I condition will make more positive interpretations post-training than the control condition. The predictor variable will be condition (CBM-I, control), the outcome variable will be interpretation bias post training. The same type of analysis will be used to test the hypothesis that the number of reported negative intrusions will be lower in the CBM-I vs. control analysis after completing the training program. T-tests will be carried out to determine if the groups differed on accuracy of comprehension questions.

Transformations
*If you plan on transforming, centering, recoding the data, or will require a coding scheme for categorical variables, please describe that process.*
The variable 'condition' (two levels: CBM-I, control) will be dummy-coded for analyses.

Follow-up analyses
*If not specified previously, will you be conducting any confirmatory analyses to follow up on effects in your statistical model, such as subgroup analyses, pairwise or complex contrasts, or follow-up tests from interactions? Remember that any analyses not specified in this research plan must be noted as exploratory.*
..

Inference criteria
*What criteria will you use to make inferences? Please describe the information you'll use (e.g. specify the p-values, Bayes factors, specific model fit indices), as well as cut-off criterion, where appropriate. Will you be using one or two tailed tests for each of your analyses? If you are comparing multiple conditions or testing multiple hypotheses, will you account for this?*
The critical p value will be set at .05. Two-tailed tests will be used.

Data exclusion
*How will you determine which data points or samples (if any) to exclude from your analyses? How will outliers be handled?*
Participants will be excluded from certain analyses if there are technical issues (e.g., data is not recorded), or if they fail to follow instructions correctly or their response accuracy falls below certain thresholds (2.5 SD below the mean). Outliers will be examined and may be excluded; skewed data will be handled by implementing bootstrapping, which does not place distributional assumptions on the data.

Appendix B

Missing data

*How will you deal with incomplete or missing data?*

While missing data is unlikely, any measures with missing data will be prorated.

Exploratory analysis

*If you plan to explore your data set to look for unexpected differences or relationships, you may describe those tests here. An exploratory test is any test where a prediction is not made up front, or there are multiple possible tests that you are going to use. A statistically significant finding in an exploratory test is a great way to form a new confirmatory hypothesis, which could be registered at a later time.*

…
